# Triterpenoids from *Vitellaria paradoxa* Stem Barks Reduce Nitrite Levels in LPS-Stimulated Macrophages

**DOI:** 10.3390/plants10051006

**Published:** 2021-05-18

**Authors:** Carmina Sirignano, Pascal Nadembega, Ferruccio Poli, Barbara Romano, Giuseppe Lucariello, Daniela Rigano, Orazio Taglialatela-Scafati

**Affiliations:** 1 Department of Pharmacy, School of Medicine and Surgery, University of Naples Federico II, Via Domenico Montesano 49, 80131 Naples, Italy; carmina.sirignano@unina.it (C.S.); barbara.romano@unina.it (B.R.); giuseppe.lucariello@unina.it (G.L.); scatagli@unina.it (O.T.-S.); 2 The UFR Life and Earth Sciences (UFR/SVT), University of Ouagadougou, Ouagadougou 848, Burkina Faso; pascal.nadembega@gmail.com; 3 Department of Pharmacy and Biotechnology, University of Bologna, Via Irnerio, 42, 40126 Bologna, Italy; ferruccio.poli@unibo.it

**Keywords:** *Vitellaria paradoxa*, triterpenes, cinnamyl esters, nitrite level reduction

## Abstract

*Vitellaria paradoxa* C. F. Gaertn is widely used in African traditional medicine as an anti-inflammatory remedy to treat rheumatism, gastric problems, diarrhea, and dysentery. The phytochemical investigation of the ethyl acetate extract of *V. paradoxa* stem bark collected in Burkina Faso led to the isolation of eight known and two triterpenes undescribed to date (**7** and **10**), in the free alcohol form or as acetyl and cinnamyl ester derivatives. The stereostructures of the new compounds were elucidated using HR-ESIMS and 1D and 2D NMR data. The isolated compounds were evaluated *in vitro* for their inhibitory effect on nitrite levels on murine macrophages J774 stimulated with the lipopolysaccharide (LPS). Among all the compounds tested, lupeol cinnamate (**3**) and betulinic acid (**5**) showed a beneficial effect in reducing nitrite levels produced after LPS stimulation.

## 1. Introduction

*Vitellaria paradoxa* (*V. paradoxa*) C. F. Gaertn (syn. *Butyrospermum parkii* (G.Don) Kotschy) is a tall (up to 14 m) tree belonging to the Sapotaceae family, occurring over nearly 1 million km^2^ within 18 African countries, but predominantly found in in the dry savannah belt of West Africa. *V. paradoxa* is strictly intertwined with the life of African populations, especially because of the largely used shea butter (Francophone usage: beurre de karité), the reason *V. paradoxa* is commonly known as the “shea butter tree”. This is an edible ivory-colored fat consisting of olein and stearin fractions along with non-saponifiable compounds, extracted by crushing and boiling the fermented kernel. It is used as an ingredient in the production of cosmetics to protect the skin in sun-protection or post-sun-exposure; to promote wound healing; to soothe skin irritation, chapping, and dermatitis; and other skin ointments [1]. The shea butter methanolic extract has been shown to exert a regulatory effect on LPS induced inflammatory responses through the inhibition of NF-κB activation, suppression of IκBα phosphorylation, and through the downregulation of mRNA and the protein expressions of pro-inflammatory cytokines and interleukins [2]. Besides shea butter, the trunk and stem barks of *V. paradoxa* macerated in traditional alcoholic beverages are widely used in traditional medicine for the treatment of pain and as an anti-inflammatory remedy to treat rheumatism, gastric problems, diarrhoea, and dysentery [1]. The methanolic stem bark extract showed significant anti-inflammatory and anti-arthritic effects in carrageenan-induced inflammation and CFA-induced arthritic animal models [3], and the stem bark ethyl acetate extract was also demonstrated to exert significant anti-inflammatory effects on adjuvant-induced arthritis with the alleviation of paw edema and hematological disorder associated with this condition [4]. Different studies have demonstrated that the anti-inflammatory activity of stem bark extracts is mainly ascribed to their rich triterpenoid composition [4], which have been proposed to act by inhibiting pro-inflammatory cytokines and iNOS and COX-2 expression [5]. Recently, a *V. paradoxa* triterpene-rich extract was shown to suppress pro-inflammatory mediators and attenuate the cartilage degradation and pain in osteoarthritis in an obesity rat model [6].

The present study aimed at obtaining a detailed phytochemical characterization of the triterpenoid composition of the stem barks of *V. paradoxa* collected in the Baskoure Area of Burkina Faso. This is a rural area located in the East-Centre region of the country, where the plant is widely used by local traditional healers, especially to treat gastrointestinal diseases, which are very common in this area [1]. The isolated compounds were assayed for their activity on reducing the nitrite level in macrophages after a lipopolysaccharide (LPS) insult, an activity related to the anti-inflammatory potential.

## 2. Results and Discussion

### 2.1. Isolation and Structural Characterization of Triterpenoids

**Figure 1:**
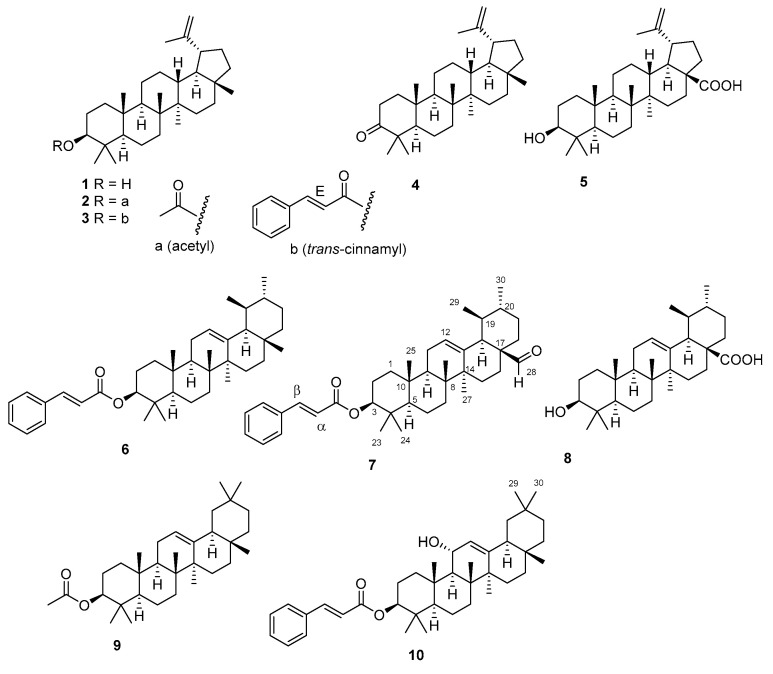
Triterpenoids characterized from stem barks of *Vitellaria paradoxa*.

Repeated purifications of the organic phase obtained from stem barks of *V. paradoxa* afforded ten pure triterpenoids (**1**–**10**; Figure 1), including the previously undescribed natural products ursaldehyde cinnamate (**7**) and 11-hydroxy-β-amyrin cinnamate (**10**). The structures of the known compounds were established as lupeol (**1**) [7], lupeol acetate (**2**) [8], lupeol cinnamate (**3**) [5], lupenone (**4**) [9], betulinic acid (**5**) [10]**,** α-amyrin cinnamate (**6**) [5], ursolic acid (**8**) [11], and β-amyrin acetate (**9**) [12] through a comparison of their spectroscopic properties with published data [5,7,8,9,10,11,12].

Ursaldehyde cinnamate (**7**) was isolated as a white powder with a molecular formula of C_39_H_54_O_3_, determined by HR-ESIMS. The ^1^H-NMR spectrum (CDCl_3_, 500 MHz) showed five methyl singlets at δ_H_ = 0.77, 0.90, 1.01, and 1.03 (6H) and two methyl doublets (δ_H_ = 0.88, *J* = 6.5 Hz, 0.95, *J* = 6.5 Hz), a pattern in agreement with the presence of a pentacyclic triterpenoid skeleton with signals characteristic of an ursolic-type skeleton. The signals in the downfield region of the ^1^H NMR spectrum were a broad double doublet at δ_H_ 4.63 (*J* = 6.0, 14.5 Hz) and a triplet at δ_H_ 5.20 (*J* = 3.3 Hz), which could be assigned to the acyl-bearing C-3 methine and to the vinylic C-12 proton, respectively. Furthermore, this region of the ^1^H-NMR spectrum showed two doublets at δ_H_ 6.45 (*J* = 15.8 Hz, Hα) and 7.67 (*J* = 15.8 Hz, Hβ), the signals of a monosubstituted benzene ring (7.39 (m, H-3′, H-4′, H-5′) and 7.53 (m, H-2′, H-6′)), all typical of a 3β-*O*-cinnamoyl moiety, and a signal at δ_H_ 9.88, clearly indicating the presence of an aldehydic function. The assignments of ^1^H and ^13^C NMR resonances in terms of structure **7** were assisted by 2D NMR measurements (COSY, HSQC, and HMBC). The C-3 methine proton resonating at δ_H_ 4.63 showed COSY cross-peaks with the geminally coupled C-2 methylene protons at δ_H_ 1.70, while the C-12 vinylic proton resonating at δ_H_ 5.20 showed vicinal couplings with the C-11 methylene protons resonating at δ_H_ 1.92. The detailed analysis of the pattern of the HMBC correlations exhibited by the seven methyl signals allowed for complete H/C assignment and confirmed the ursolic-type structure with an aldehydic function at C-28 (HMBC cross-peak H-18/C-28). The cross peak between H-3 (δ_H_ 4.63) and the cinnamate ester carbonyl (δ_C_ 166.9), completed the definition of the structure of compound **7**. Proton and carbon resonances, as well as proton–proton coupling constants assigned to **7** almost exactly paralleled those reported for ursaldehyde acetate, differing from **7** only for the acylating group [13]. In particular, the chemical shift and coupling constants measured for the C-3 methine indicated an equatorial (β) orientation of the acyl group.

Compound **10** was isolated as a white amorphous powder with a molecular formula of C_39_H_56_O_3_, determined by HR-ESIMS. An analysis of the 1D NMR signals was carried out by extensive application of 2D NMR spectroscopy (COSY, HSQC, HMBC), which yielded to the assignment of **10** as a close analogue of β-amyrin cinnamate [5], previously characterized from shea fat. The single difference between the two series of signals were located in the downfield region of the spectrum, where compound **10** showed the presence of one additional oxymethine signal (δ_H_ 4.55, H-11) and the downfield shift of the H-12 (δ_H_ 5.36). The COSY cross-peaks between δ_H_ 4.55 and δ_H_ 5.36 confirmed the location of the additional hydroxy group at C-11, whose α-orientation was assigned on the basis of the H-9/H-11 coupling constant (*J* = 10 Hz). A further support to the stereostructure assignment of compound **10** came from a comparison of the ^1^H/^13^C NMR data and proton–proton coupling constants with those reported for 11α-hydroxy-β-amyrin isolated from *Stautonia hexaphylla* [14].

Triterpene alcohols such as α-amyrin, β-amyrin, lupeol, and butyrospermol, most of which occur as acetyl and cinnamyl ester derivatives, have been previously reported to be the main non-glyceride constituents of *V. paradoxa* kernel [5,6,15,16], leaves [17], and stem bark [4,10,18] extracts. Typically, the kernel fat contains ca. 0.5–6.5% of triterpene esters depending on the geographic site; for instance, shea samples from the western area of the shea belt (Cote d’Ivoire, Ghana, and Nigeria) and from Cameroun generally contain highest percentages of these compounds (2.0–6.5%) [19]. Some of these metabolites exhibited different biological properties such as cytotoxicity [10,18], a significant *in vitro* and *in vivo* antitrypanosomal activity [17], inhibitory effects on TPA-induced inflammation in mice and on skin tumor promotion in an in vivo two-stage carcinogenesis test [5], and other bioactivities demonstrating the bio-pharmacological potential of these compounds [16].

### 2.2. Biological Activity of Triterpenoids

**Figure 2:**
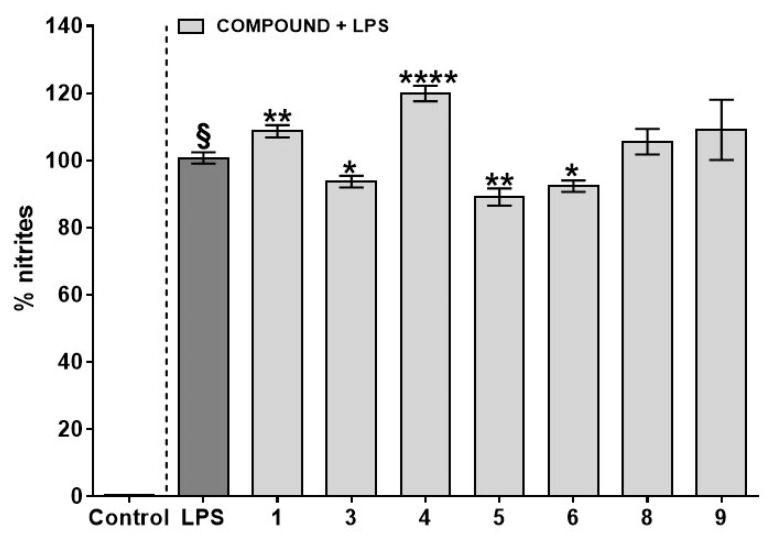
Effects of *V. paradoxa* selected triterpenoids (compounds **1**, **3**–**6**, and **8**–**9**) on nitrite levels in the supernatant collected from J774A.1 murine macrophages stimulated with lipopolysaccharide (LPS, 1 μg/mL) for 24 h. The compounds used in this study were supplemented to the cell media 30 min before LPS stimulus. Results are reported as percentage of nitrites and are expressed as mean ± SEM of four independent experiments (in quadruplicate). § *p* < 0.0001 vs. control (cells without LPS); * *p* < 0.05, ** *p* < 0.01, and **** *p* < 0.0001 vs. LPS as assessed by one-way ANOVA, followed by Dunnett’s multiple comparisons test.

Given the remarkable anti-inflammatory action of stem bark extracts [3,4,5,6,19], we initially tested the effect of seven pure compounds among those isolated in this study (compounds **7** and **10** were not tested because of the low amount available, while **2** underwent degradation during tests) on the nitrite levels produced in response to LPS stimulus in murine macrophages J774A.1 Among all of the compounds tested, lupeol cinnamate (**3**), betulinic acid (**5**), and α-amyrin cinnamate (**6**) at 10 µM showed a beneficial effect in reducing the nitrite levels produced after LPS stimulation (Figure 2). Because of availability reasons, compounds **3** and **5** were selected for further pharmacological studies. Of note, at a 30 µM concentration, none of the tested compounds affected the cell viability after 24 h of treatment, thus excluding the likelihood that their effect in reducing nitrite levels may be because of a non-specific cytotoxic effect in macrophages (data not shown).

**Figure 3:**
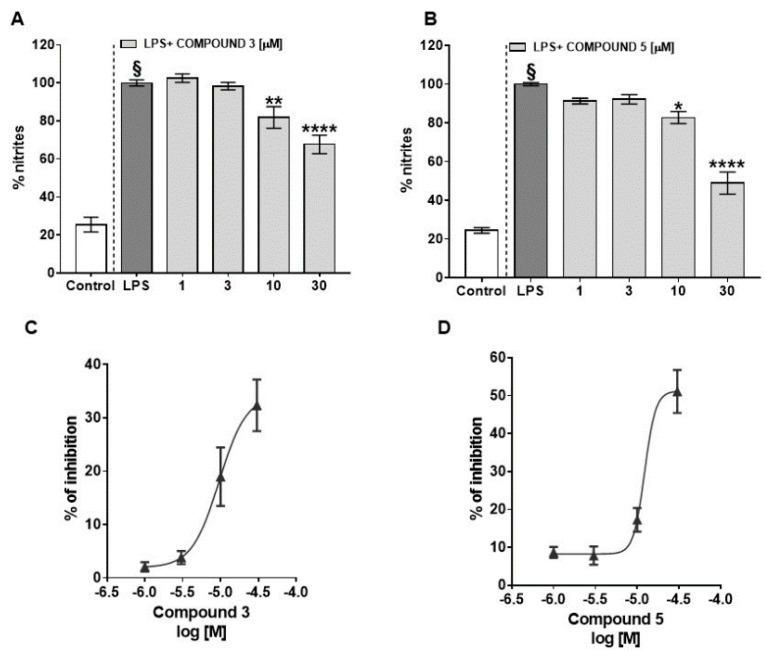
Effects of lupeol cinnamate (compound **3**) (**A**) and betulinic acid (compound **5**) (**B**) on nitrite levels produced in the cell medium of J774A.1 murine macrophages in response to lipopolysaccharide (LPS; 1 μg/mL) for 24 h. Compounds **3** and **5** were given to the cell media 30 min before LPS insult. Results are reported as percentage of nitrites and expressed as mean ± SEM of three or four independent experiments. § *p* < 0.0001 vs. control (cells without LPS); * *p* < 0.05, ** *p* < 0.01, and **** *p* < 0.0001 vs. LPS as assessed by one-way ANOVA, followed by Dunnett’s multiple comparisons test. IC_50_ curves (concentration of the compounds that caused the 50% inhibition of nitrite production) for lupeol cinnamate (compound **3**) (**C**) and betulinic acid (compound **5**) (**D**) were calculated by non-linear regression analysis using the sigmoid concentration–response curve.

LPS stimulus (1 μg/mL) given for 24 h to murine macrophages determined an increase in nitrite levels, while a pre-treatment (1–30 μM 30 min before LPS) with lupeol cinnamate (**3**) (Figure 3A) and/or betulinic acid (**5**) (Figure 3B), significantly and in a concentration dependent manner, lowered LPS-induced nitrite production. The IC_50_ (concentration that caused the 50% inhibition of nitrite production) calculated for these compounds were 9.5 ± 0.11 μM for lupeol cinnamate (**3**) (Figure 3C) and 12.2 ± 0.11 μM for betulinic acid (**5**) (Figure 3D).

**Figure 4:**
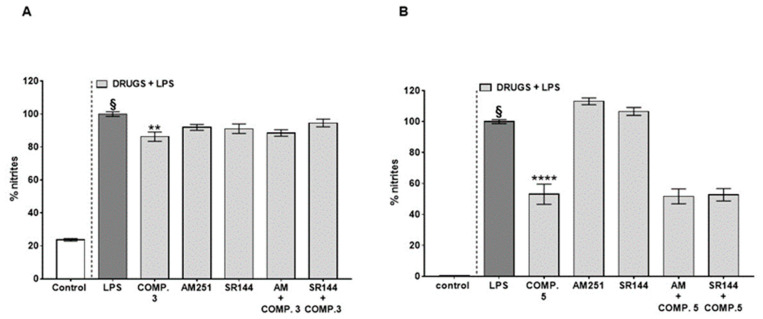
Effect of lupeol cinnamate (compound **3**) (**A**) and betulinic acid (compound **5**) (**B**) on nitrite levels produced in response to lipopolysaccharide (LPS, 1 μg/mL) added to J774A.1 murine macrophages for 24 h alone or in the presence of the selective cannabinoid CB1 receptor antagonist AM251 (1 μM) and/or in presence of the selective cannabinoid CB2 receptor antagonist SR144528 (SR144, 0.1 μM). Treatment with antagonists started 30 min before exposure to compounds **3** and/or **5**. LPS (1 μg/mL) was given 30 min after the administration of the drugs (antagonists, compounds **3** and **5**). Results are reported as a percentage of nitrites and are expressed as means ± SEM of three or four independent experiments. § *p* < 0.0001 vs. control (cells without LPS); ** *p* < 0.01 and **** *p* < 0.0001 vs. LPS as assessed by one-way ANOVA, followed by Tukey’s multiple comparisons test.

As some triterpenoids, including β-amyrin, have been demonstrated to exert analgesic and anti-inflammatory pharmacological effects via indirect cannabimimetic mechanisms by inhibiting the degradation of the endocannabinoid 2-arachidonoylglycerol [20], we tested the active compounds in presence of AM251 (selective CB_1_ receptor antagonist) or SR144528 (selective CB_2_ receptor antagonist), but no significant change in the activity was detected when co-administered with lupeol cinnamate (**3**) (Figure 4A) and/or betulinic acid (**5**) (Figure 4B). AM251 and SR144528, at the concentrations used, did not alter the nitrite levels produced in response to LPS (Figure 4A,B).

The selective activity shown by compounds **3**, **5**, and **6** points to the existence of structural requirements that are not easy to unveil from the limited number of compounds tested. Compared with **1** and **4**, the presence of a cinnamate ester in **3** seems to be beneficial for the bioactivity. This is confirmed by previous studies, which show that esterifications at 3-OH of pentacyclic triterpenes seem to enhance the antinflammatory activity [5]. It is also interesting to note the higher activity of betulinic acid compared with ursolic acid, clearly evidencing that details in the triterpene skeleton can have a major impact on the activity.

## 3. Materials and Methods

### 3.1. General Experimental Procedures

Optical rotations (CHCl_3_) were measured at 589 nm on a P2000 Jasco polarimeter. The ^1^H (500 MHz) and ^13^C (125 MHz) NMR spectra were measured at room temperature on a Varian INOVA 500 MHz spectrometer. Chemical shifts were referenced to the residual solvent signal (CDCl_3_: δH 7.26, δC 77.0). Homonuclear ^1^H connectivities were determined using the COSY experiment, one-bond heteronuclear ^1^H-^13^C connectivities were determined using the HSQC experiment, and two- and three-bond ^1^H-^13^C connectivities were determined using gradient-HMBC experiments optimized for a ^2,3^*J* of 8 Hz. Low- and high-resolution ESI-MS spectra were performed on a LTQ OrbitrapXL (Thermo Scientific) mass spectrometer with a triple quadrupole analyzer. Medium-pressure liquid chromatography (MPLC) was performed on a Büchi (Switzerland) apparatus using a silica gel (7–230 mesh). Separations were monitored by TLC on Merck 60 F254 silica gel (0.25 mm) plates; HPLC was achieved on a Knauer 1800 instrument equipped with a refractive index detector. LUNA (normal phase, SI60, or reverse-phase RP18, 250 × 4 mm; Phenomenex) columns were used, with 1 mL/min as the flow rate, with isocratic elution at room temperature (see Section 3.3 for solvents).

### 3.2. Plant Material

The stem barks of *Vitellaria paradoxa* were collected in the summer of 2008 in Baskoure and Songretenga rural areas located in Kourittenga Province, in the East-Centre Region of Burkina Faso, as described in the literature [1]. The plant identification was done by Prof. Joseph Issaka Boussim (University of Ouagadougou), and voucher specimens (V-08-01) were deposited into the herbarium of the Botanical Laboratory of the University of Ouagadougou.

### 3.3. Chromatographic Purification

*Vitellaria paradoxa* stem bark (250 g) was dried in the shade at room temperature, and was powdered and extracted exhaustively by maceration in methanol (3 × 6 L) to afford 50 g of a crude extract after the removal of the solvent under a vacuum. The methanol extract was suspended in water and then partitioned in sequence against EtOAc and *n*-butanol to yield 18 g and 27 g of organic phases, respectively. The EtOAc extract (18 g) was subjected to MPLC over a silica gel column (230–400 mesh) and eluted with a solvent gradient of increasing polarity from *n*-hexane to EtOAc, EtOAc−MeOH (1:1), and finally MeOH. Altogether, 13 fractions were obtained, which were then further purified by normal and reverse-phase HPLC. Fraction 4 (3.8 g) eluted with *n*-hexane-EtOAc, 95:5, was separated by normal phase HPLC (*n*-hexane/EtOAc 95:5, flow rate 1.0 mL/min) to afford α-amyrin cinnamate (6, 3.3 mg, >95% purity by HPLC), lupeol cinnamate (3, 142.7 mg, >95% purity by HPLC), lupeol acetate (2, 11 mg, >98% purity by HPLC), lupenone (4, 20.4 mg, >99% purity by HPLC), and ursaldehyde cinnamate (7, 1.0 mg, >95% purity by HPLC). Fraction 5 (330 mg), eluted with *n*-hexane-EtOAc, 85:15, was separated by normal phase HPLC (*n*-hexane/EtOAc 85:15, flow rate 1.0 mL/min) to afford lupeol (1, 8.1 mg, >95% purity by HPLC). This fraction was further purified by reverse phase HPLC (MeOH/H_2_O 95:5, flow rate 1.0 mL/min) to afford 11-OH β-amyrin cinnamate (10, 1.0 mg, >95% purity by HPLC). Fraction 9 (240 mg), eluted with *n*-hexane-EtOAc, 70:30, was separated by HPLC (*n*-hexane-EtOAc, 7:3, flow rate 1.0 mL/min) to obtain β-amyrin acetate (9, 3.0 mg, >95% purity by HPLC), betulinic acid (5, 10 mg, >95% purity by HPLC), and ursolic acid (8, 5.3 mg, >95% purity by HPLC) in their pure form.

#### 3.3.1. Ursaldehyde Cinnamate (7)

Amorphous powder. ^1^H NMR (CDCl_3_, 500 MHz): δ_H_ 0.77 (3H, s, H-24), 0.79 (1H, br d, *J* = 12.1 Hz, H-5), 0.88 (3H, d, *J* = 6.5 Hz, H-29), 0.90 (3H, s, H-23), 0.91 (1H, m, H-19), 0.95 (3H, d, *J* = 6.5 Hz, H-30), 0.98 (1H, m, H-15a), 1.01 (3H, s, H-25), 1.03 (3H, s, H-26), 1.06 (3H, s, H-27), 1.13 (1H, m, H-1a), 1.25 (1H, m, H-21a), 1.27 (1H, m, H-22a), 1.31 (1H, m, H-20), 1.33 (1H, m, H-7a), 1.38 (1H, m, H-6a), 1.39 (1H, m, H-21b), 1.42 (1H, m, H-22b), 1.44 (1H, m, H-16a), 1.53 (1H, m, H-6b), 1.55 (1H, m, H-7b), 1.57 (1H, m, H-9), 1.67 (1H, m, H-1b), 1.70 (2H, m, H-2), 1.82 (1H, m, H-15b), 1.92 (2H, dd, *J* = 3.6 and 7.0 Hz, H-11), 1.98 (1H, d, *J* = 10 Hz, H-18), 2.00 (1H, m, H-16b), 4.63 (1H, br dd, *J* = 6.0 and 14.5 Hz, H-3), 5.20 (l H, t, *J* = 3.3 Hz, H-12), 6.45 (1H, d, *J* = 15.8 Hz, Hα), 7.39 (3H, m, *J* = 3.6, 8.6 Hz, H-3′,4′,5′), 7.53 (2H, m, *J* = 3.6, 8.6 Hz, H-2′,6′), 7.67 (1H, d, *J* = 15.8 Hz, Hβ), 9.88 (l H, d, *J* = 1.2 Hz, H-28). ^13^C NMR (CDCl_3_, 125 MHz): δ_C_ 15.7 (C-25), 16.5 (C-26), 16.9 (C-24), 17.5 (C-29), 18.2 (C-6), 21.4 (C-30), 23.2 (C-27), 23.4 (C-11), 23.6 (C-2), 26.7 (C-15), 28.2 (C-16), 28.8 (C-28), 29.1 (C-23), 31.2 (C-21), 32.1 (C-22), 32.9 (C-7), 36.8 (C-10), 37.9 (C-4), 38.5.3 (C-1), 39.6 (C-19), 39.7 (C-20), 40.0 (C-8), 42.0 (C-14), 47.6 (C-9), 50.4 (C-17), 55.2 (C-5), 59.0 (C-18), 81.0 (C-3), 118.6 (C-α), 126.3 (C-12), 128.1 (C-2′), 128.1 (C-6′), 129.0 (C-3′), 129.0 (C-5′), 130.2 (C-4′), 134.3 (C-1′), 139.6 (C-13), 144.2 (C-β), 166.9 (C=O), and 207.7 (C-28). ESIMS (positive ions) *m/z* 593 [M + Na]^+^; HR-ESIMS *m/z* 593.3982; calculated for C_39_H_54_O_3_Na *m/z* 593.3971.

#### 3.3.2. 11-Hydroxy-β-Amyrin Cinnamate (10)

Amorphous powder. ^1^H NMR (CDCl_3_, 500 MHz): δ_H_ 0.81 (3H, s, H-28), 0.91 (3H, s, H-30), 0.92 (3H, s, H-29), 0.89 (1H, br d, *J* = 6 Hz, H-5), 0.90 (3H, s, H-23), 0.93 (3H, s, H-24), 0.95 (1H, m, H-15a), 0.97 (3H, s, H-26), 0.98 (3H, s, H-25), 1.01 (1H, m, H-19a), 1.09 (1H, m, H-1a), 1.14 (1H, m, H-21a), 1.17 (3H, s, H-27), 1.26 (1H, m, H-22a), 1.32 (1H, m, H-7a), 1.34 (1H, m, H-21b), 1.40 (1H, m, H-6a), 1.41 (1H, m, H-22b), 1.43 (1H, m, H-16a), 1.54 (1H, m, H-7b), 1.55 (1H, m, H-6b), 1.65 (1H, m, H-1b), 1.57 (1H, d, *J* = 10.0 Hz,, H-9), 1.73 (1H, m, H-15b), 1.65 (1H, m, H-19b), 1.69 (2H, m, H-2), 2.05 (1H, d, *J* = 10 Hz, H-18), 2.10 (1H, m, H-16b), 4.55 (1H, dd, *J* = 3.0, 10.0 Hz, H-11), 4.68 (1H, t, *J* = 8, H-3), 5.36 (1H, d, *J* = 3.0 Hz, H-12), 6.44 (1H, d, *J* = 15.8 Hz, Hα), 7.39 (3H, m, *J* = 3.6, 8.6 Hz, H-3′,4′,5′), 7.53 (2H, m, *J* = 3.6, 8.6 Hz, H-2′,6′), 7.67 (1H, d, *J* = 15.8 Hz, Hβ). ^13^C NMR (CDCl_3_, 125 MHz): δ_C_ 15.7 (C-25), 16.8 (C-26), 16.9 (C-24), 18.2 (C-6), 23.6 (C-2), 23.7 (C-30), 25.8 (C-27), 26.4 (C-15), 27.2 (C-16), 28.1 (C-23), 28.8 (C-28), 31.7 (C-20), 32.4 (C-17), 32.9 (C-7), 33.3 (C-29), 34.2 (C-21), 37.1 (C-22), 37.9 (C-4), 38.5 (C-1), 38.8 (C-10), 42.0 (C-14), 43.0 (C-8), 47.0 (C-18), 46.6 (C-19), 47.6 (C-9), 55.2 (C-5), 82.0 (C-11), 81.0 (C-3), 118.6 (C-α), 121.6 (C-12), 128.1 (C-2′), 128.1 (C-6′), 129.0 (C-3′), 129.0 (C-5′), 130.2 (C-4′), 134.3 (C-1′), 144.2 (C-β), 151.6 (C-13), and 166.9 (C=O). ESIMS (positive ions) *m/z* 595 [M + Na]^+^; HR-ESIMS *m/z* 595.4122; calculated for C_39_H_56_O_3_Na *m/z* 595.4127.

### 3.4. Pharmacological Evaluation

#### 3.4.1. Cell Culture

J774A.1 murine macrophages (ATCC, from LGC Standards, Milan, Italy) were used for the pharmacological evaluation of the compounds. The cells were routinely maintained at 37 °C in a humidified incubator with 5% CO_2_ and were cultured in Dulbecco’s modified Eagle’s medium (DMEM, Lonza Group), supplemented with 10% fetal bovine serum (FBS), 100 U/mL penicillin and 100 µg/L streptomycin, 2 mM L-glutamine, 20 mM Hepes (4-(2-hydroxyethyl)-1-piperazineethanesulphonic acid), and 1 mM sodium pyruvate. The medium was changed every 48 h, following manufacturer’s protocols, and cell viability was evaluated by trypan blue exclusion.

#### 3.4.2. Nitrite Measurement and Pharmacological Treatment In Vitro

The anti-inflammatory effect of compounds **1**, **3**–**5**, **8**–**9** (10 μM), and/or compounds **3** and **5** (1–30 μM) was evaluated by measuring nitrite and stable metabolites of nitric oxide (NO) in a macrophage medium via colorimetric assay, as previously described [21]. J774A.1 murine macrophages were plated in 24-well plates (2.5 × 10^5^ cells per well) and treated with compounds **1**, **3**–**5**, **8**–**9** (10 μM), and/or compounds **3** and **5** (1–30 μM) for 30 min before LPS insult (1 µg/mL) for 24 h. Dexamethasone (1 μM), used as a positive control, determined the inhibitory effects on the nitrite levels ranging from 42.19% to 47.96%. Using this assay, the effect of compounds **3** and/or **5** on nitrite production was also evaluated in the presence of AM251 (1 μM, CB_1_ selective antagonist) and SR144528 (0.1 μM, CB_2_ selective antagonist; all from Tocris, Italy) added to the cell media 30 min before compound **3** (10 μM) and/or compound **5** (30 μM). Then, the supernatants were collected and incubated with 100 µL of Griess reagent (0.2% naphthylethylenediamine dihydrochloride and 2% sulphanilamide in 5% phosphoric acid) at room temperature for 10 min, in order to enable the formation of a colored azo dye. The absorbance was read at 550 nm on a Thermo Scientific Multiskan GO instrument. The absorbance values were compared to a serial-diluted sodium nitrite (Sigma-Aldrich) that was used as a standard curve. The data were expressed as a percentage of the nitrite production inhibition (*n* = 4 independent experiments, including three to four replicates for each treatment).

#### 3.4.3. Statistical Analysis

Data are expressed as mean ± SEM of n experiments. To determine statistical significance, one-way ANOVA followed by Tukey’s multiple comparisons test and/or Dunnett’s multiple comparisons test was used for the comparison of multiple groups. The IC_50_ (concentration that caused 50% inhibition of nitrite production) ±95% confidence interval values were calculated by non-linear regression analysis using the sigmoid concentration– response curve equation (GraphPad Prism 7).

## 4. Conclusions

In conclusion, a detailed phytochemical investigation on the stem barks obtained from the African plant *V. paradoxa* resulted in the isolation of ten triterpenoids belonging to three different pentacyclic structural families, namely lupane, ursane, and oleanane classes. Among these compounds, we characterized two unprecedented triterpenoids, the ursane ursaldehyde cinnamate (**7**) and the oleanane 11-hydroxy-β-amyrin cinnamate (**10**). Three of the isolated compounds induced a reduction in nitrite levels in LPS-stimulated macrophages, an activity related to the anti-inflammatory potential. These results suggest the possible response of the anti-inflammatory properties demonstrated for the stem bark extract [4], although a synergistic action of the complex triterpenoid mixture is likely.

## Data Availability

Data is contained within the article or supplementary material. Further data is available on request.

## Supplementary Materials

The following are available online at https://www.mdpi.com/article/10.3390/plants10051006/s1, Figure S1: Negative ion MS of lupeol; Figure S2: Positive ion MS of lupeol acetate; Figure S3: Positive ion MS of lupeol cinnamate; Figure S4: Negative ion MS of lupenone; Figure S5: Negative ion MS of betulinic acid; Figure S6: Positive ion MS of α-amyrin cinnamate; Figure S7: Positive ion MS of ursaldehyde cinnamate; Figure S8: Negative ion MS of ursolic acid; Figure S9: Positive ion MS of β-amyrin acetate; Figure S10: Negative ion MS of 11-hydroxy-β-amyrin cinnamate.
