# Supplementary material for: Triterpenoids from Vitellaria paradoxa Stem Barks Reduce Nitrite Levels in LPS-Stimulated Macrophages
Source: Plants (Basel). 2021 May 18;10(5):1006. doi: 10.3390/plants10051006 (PMC8158121; doi:10.3390/plants10051006)
Supplement: Supplementary file 1 [file plants-10-01006-s001.zip › plants-1210074-supplementary.pdf]

SUPPLEMENTARY MATERIAL

Triterpenoids from *Vitellaria paradoxa* Stem Barks Reduce Nitrite Levels in LPS-Stimulated Macrophages

Carmina Sirignano [1], Pascal Nadembega [2], Ferruccio Poli [3], Barbara Romano [1], Giuseppe Lucariello [1], Daniela Rigano [1,*], and Orazio Taglialatela-Scafati [1]

[1] Department of Pharmacy, School of Medicine and Surgery, University of Naples Federico II, Via Domenico Montesano 49, 80131 Naples, Italy.; carmina.sirignano@unina.it (C.S.); barbara.romano@unina.it (B.R.); giuseppe.lucariello@unina.it (G.L.); drigano@unina.it (D.R.); scatagli@unina.it (O.T.S.)

[2] University of Ouagadougou UFR/SVT, 03 BP, 848 Ouagadougou 03, Burkina Faso; pascal.nadembega@gmail.com

[3] Department of Pharmacy and Biotechnology, University of Bologna, Via Irnerio, 42, 40126, Bologna, Italy; ferruccio.poli@unibo.it

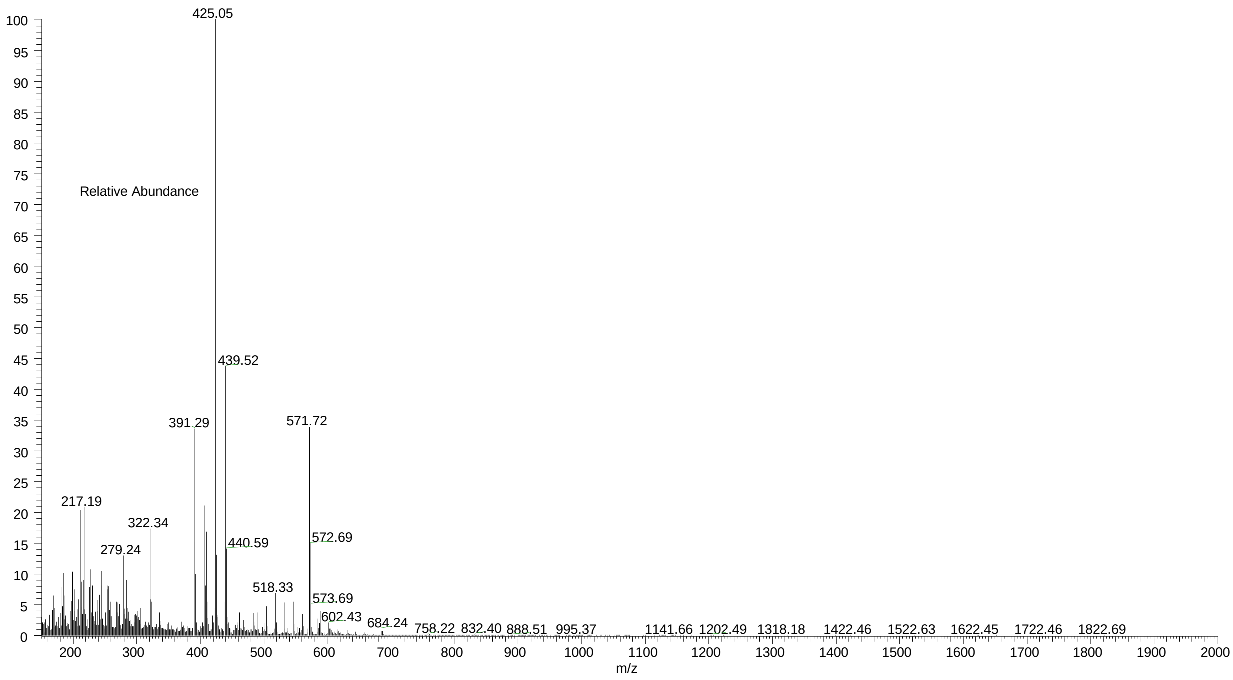

Figure S1. Negative ion MS of lupeol (**1**)

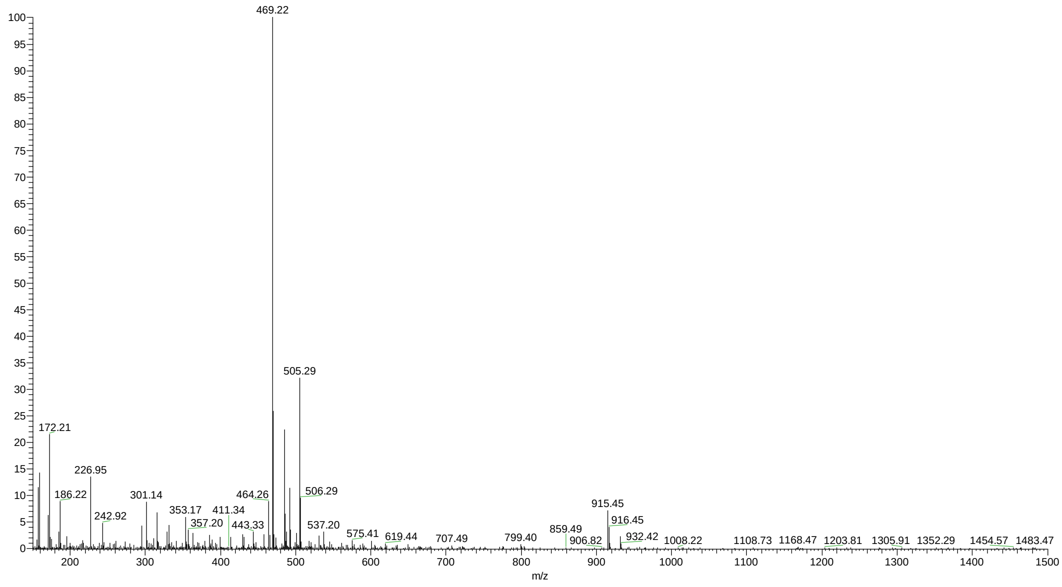

Figure S2. Positive ion MS of lupeol acetate (**2**)

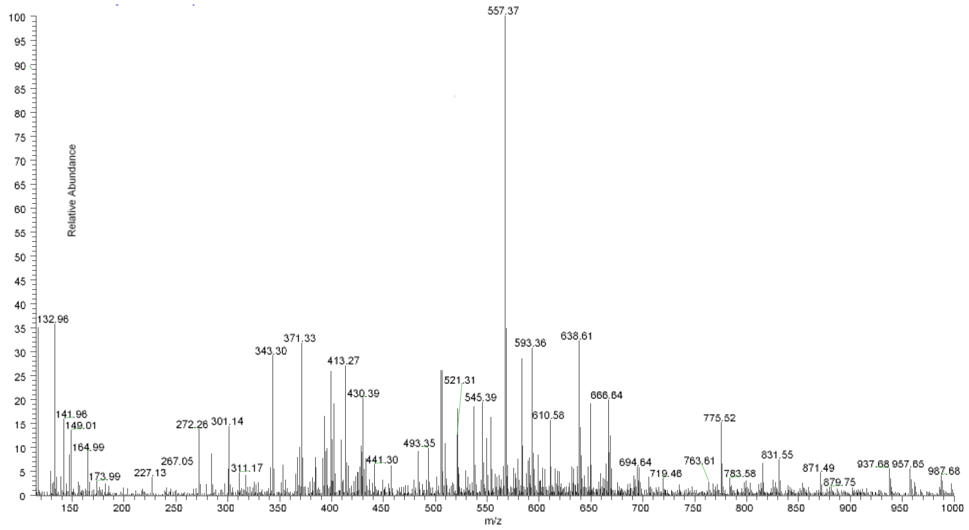

Figure S3. Positive ion MS of lupeol cinnamate (**3**)

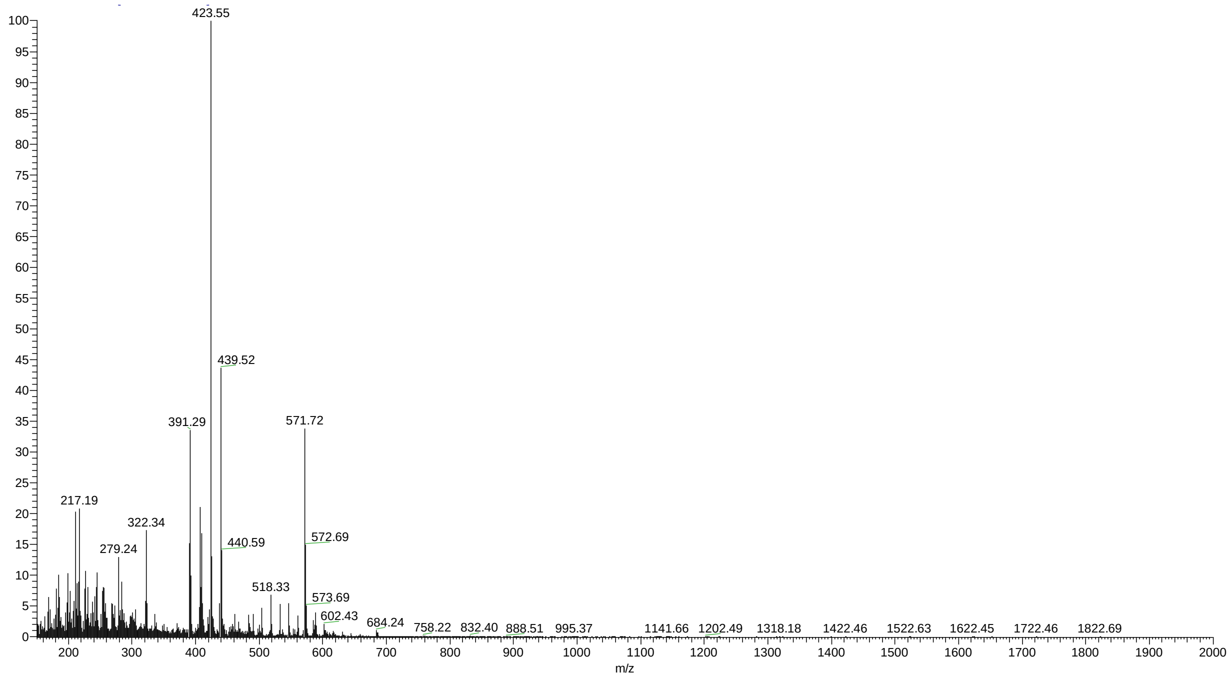

Figure S4. Negative ion MS of lupenone (**4**)

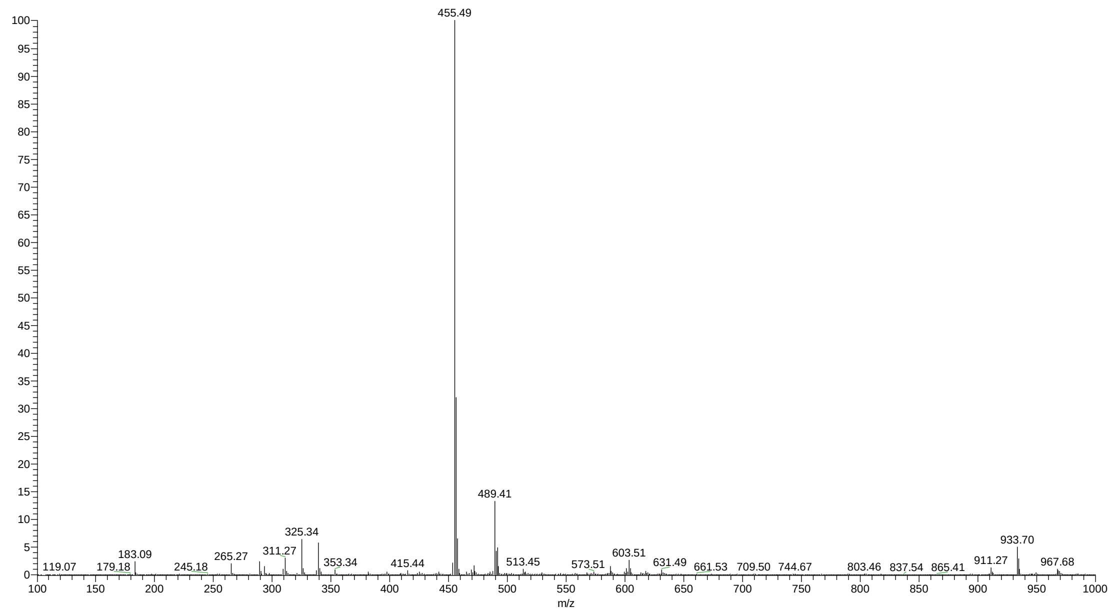

Figure S5. Negative ion MS of betulinic acid (**5**)

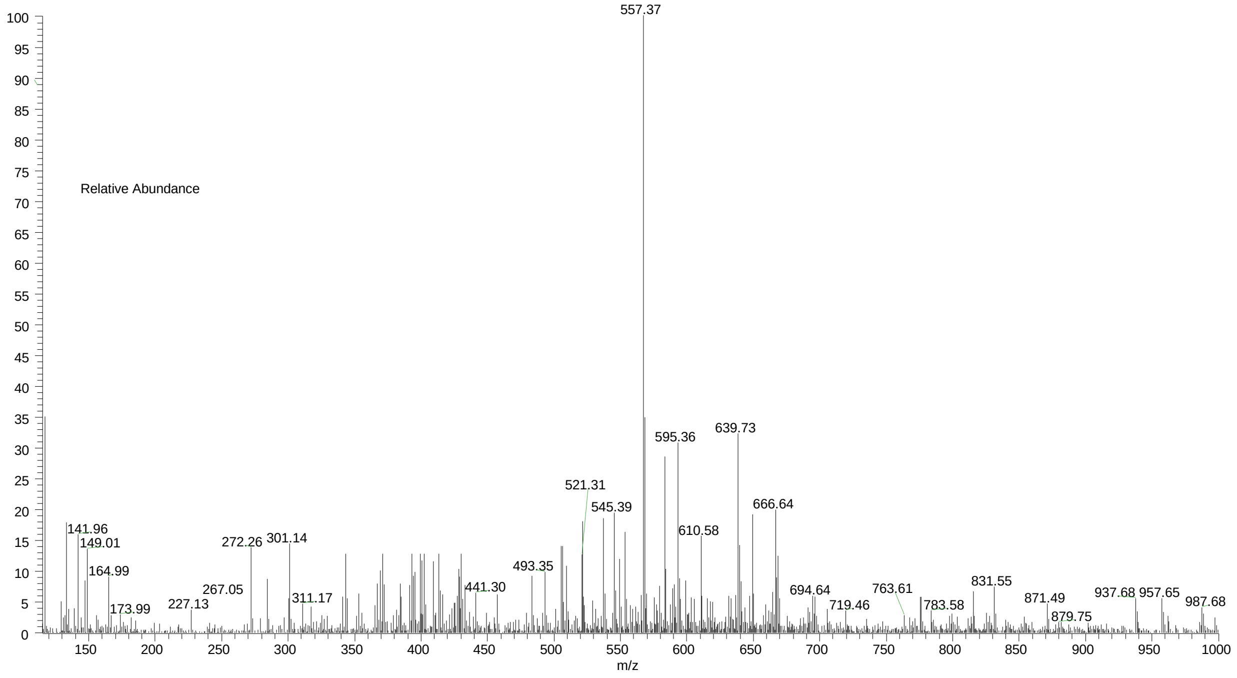

Figure S6. Positive ion MS of α-amyrin cinnamate (**6**)

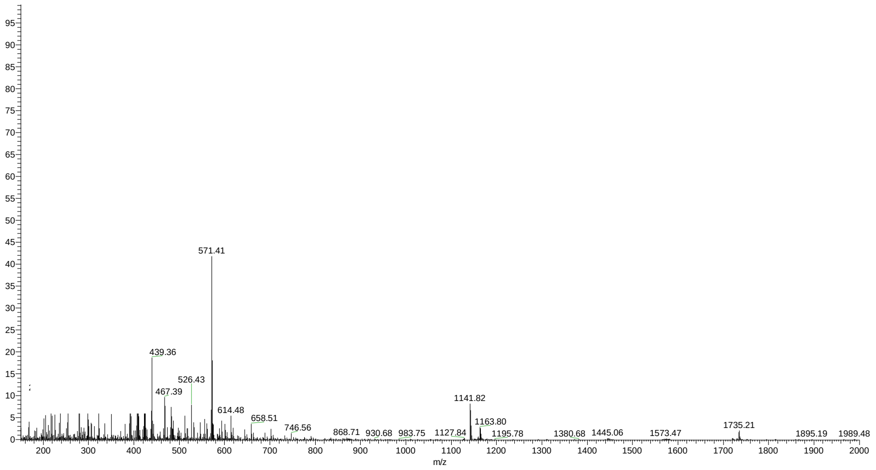

Figure S7. Positive ion MS of ursaldehyde cinnamate (**7**)

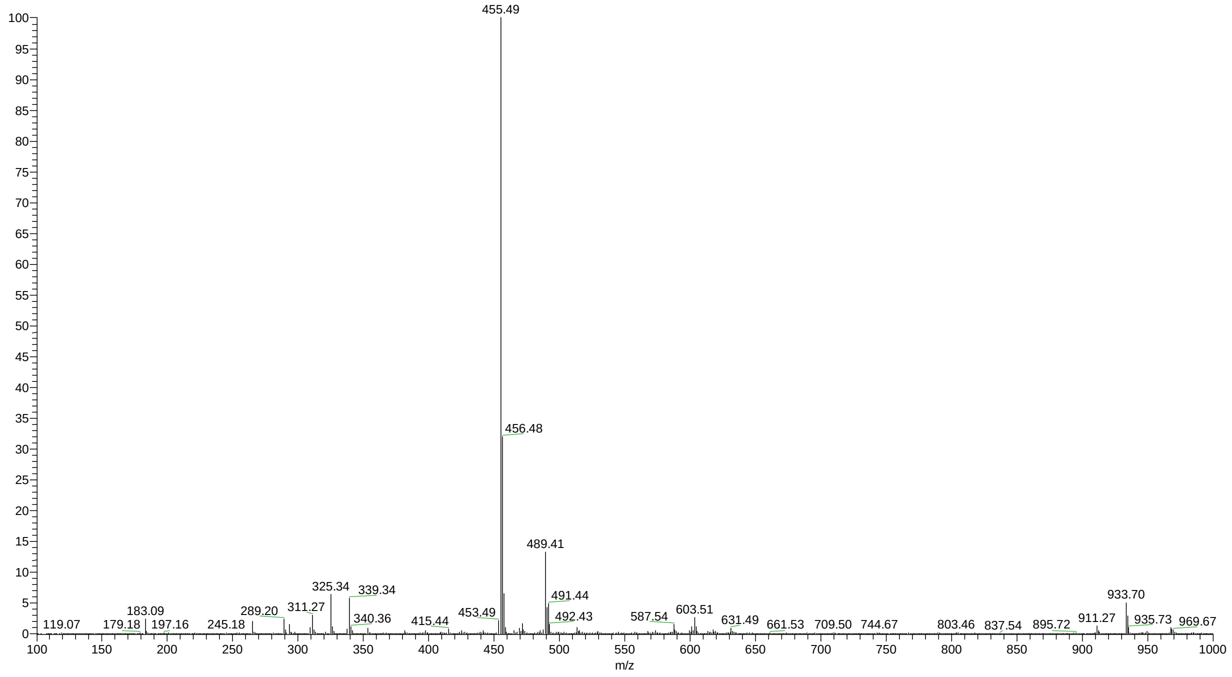

Figure S8. Negative ion MS of ursolic acid (**8**)

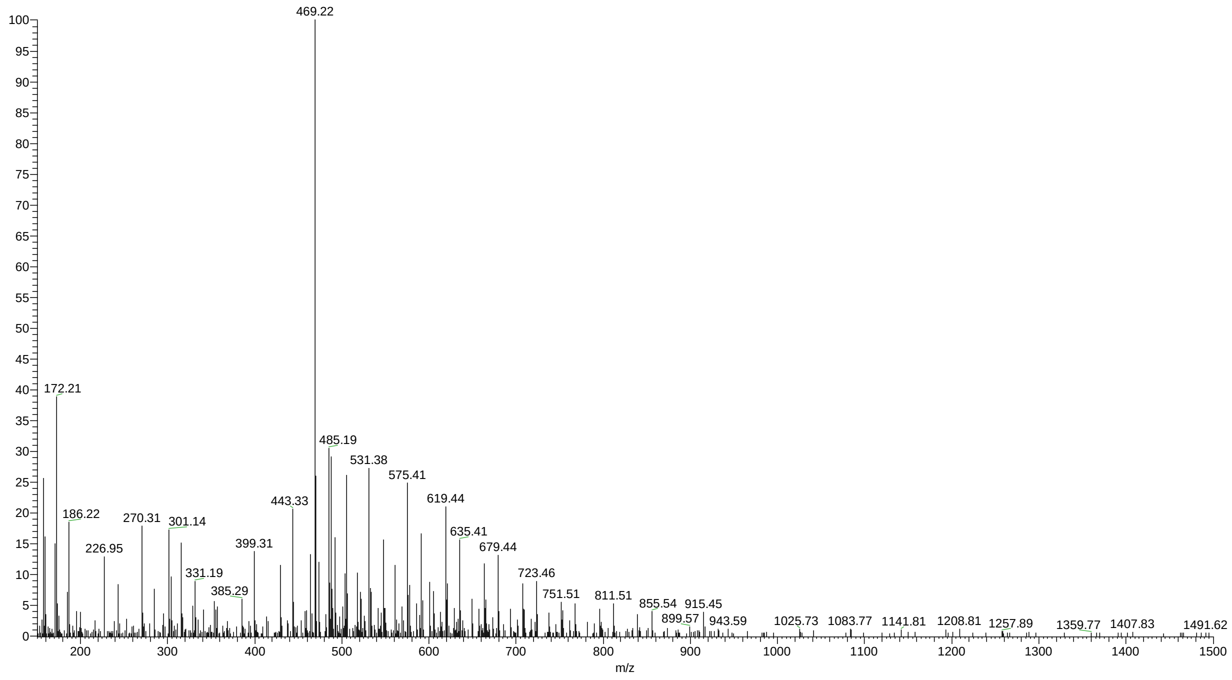

Figure S9. Positive ion MS of β-amyrin acetate (**9**)

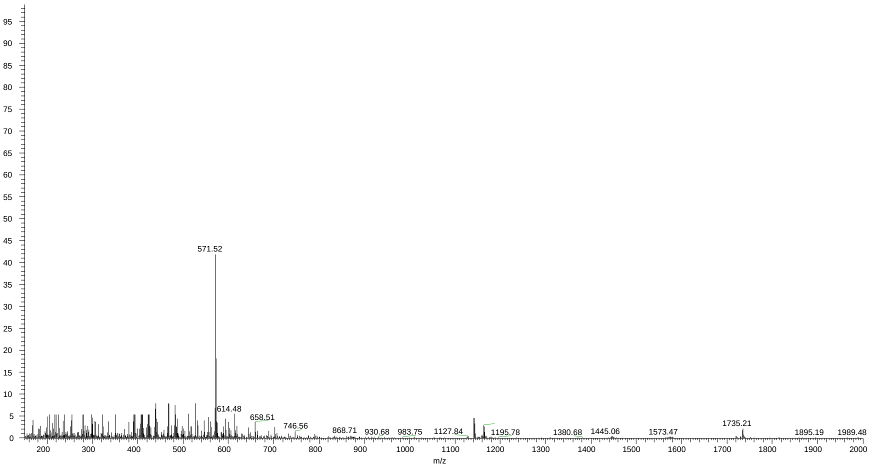

Figure S10. Negative ion MS of 11-hydroxy-β-amyrin cinnamate (**10**)
